# Gender preferences for multiple attributes of soil and water conservation in Northern Rwanda

**DOI:** 10.1016/j.heliyon.2024.e35518

**Published:** 2024-08-05

**Authors:** Ildephonse Musafili, Oscar Ingasia Ayuya, Eliud Abucheli Birachi

**Affiliations:** a Department of Agricultural Economics and Agribusiness Management, Egerton University, Kenya; b International Center for Tropical Agriculture (CIAT), Rwanda

**Keywords:** Gender preferences, SWC attributes, Best–worst experiment, Northern Rwanda

## Abstract

Despite the dominance of female labor in agricultural production, female-controlled farm plots have lower efficiency compared to plot managed by male-headed households, which indicates a huge gender disparity in agricultural productivity. Overlooking gender preferences when designing interventions that promote the conservation of soil and water resources might face challenges in adoption and could result in ineffective policies to close the gender gap. This study seeks to analyze gender-specific preferences regarding attributes of soil and water conservation (SWC) in northern Rwanda. A best-worst survey was conducted among 653 respondents, comprising 253 males and 400 females, representing 422 households surveyed between September and December 2019. The analysis of BWS data involved assessing attribute-level relative importance, Pearson correlation, and maximum difference scaling using multinomial logit (MNL). Findings from attribute-level importance analysis revealed significant gender-based disparities in preferences across three important SWC attribute scenarios: the high scenario (between 65 % and 100 %), the moderate scenario (between 50 % and 65 %), and the basic scenario (with <50 % relative importance). The study identified heterogeneity in preferences regarding the relative importance of SWC attribute levels. Pearson correlation analysis revealed substantial synergies among attribute levels linked to land consolidation, improved land tenure, and joint SWC decision-making between genders. Additionally, the study identified trade-offs among multiple levels of SWC attributes, including households' SWC decision-making and physical and structural measures. The results from MNL regression show that both males and females exhibit positive preferences for multiple levels of SWC attributes, but show negative preferences when it comes to household decisions involving multiple SWC strategies. The study highlights the importance of equal opportunities for males and females’ participation in agricultural transformation through the adoption of SWC technologies as a fundamental step towards sustainable agricultural intensification. It advocates for gender transformational approaches to incentivize the scaling up of SWC practices and promote packages with lower uptake rates. Additionally, the study suggests enhancing knowledge and extension education in SWC to better understand diverse needs and preferences of female farmers.

## 1 Introduction

Gender has been globally viewed as a key dimension in agricultural development strategies through increasing food production and promoting economic development [1]. Within the sub Saharan African region, gender dynamics in agriculture are characterized by two features: the predominance of female labor in agricultural production, indicating higher female representation compared to men, and a notable gender gap in agricultural productivity, whereby farms owned by female-headed households generally exhibit lower production efficiency than those owned by male-headed households [2,3,4]. The discrepancies could be clarified through male and female differences in embracing yield-enhancing and soil-restoring technologies. Furthermore, the gap in agricultural productivity is shaped by structural disadvantages specific to females (socioeconomic and environmental), different utilization of inputs and female labor in farming techniques, and inherent endowment factors [5,6]. Understanding gender specific preferences is crucial for formulating effective policies that improve access to productive resources and intra-household decision-making regarding SWC. This understanding can greatly increase the adoption of soil and water conservation (SWC) technologies, leading sustainable enhancements in agricultural productivity [7].

Gender disparities in resource control and negotiation within households also contribute to the gender gaps observed in agricultural productivity [8,9]. Moreover, enduring disparities in income diversification give rise to productivity gaps and variances in human capital [10]. [11] argued that the disparity in gender does not result from a lack of efficiency among women farmers but rather from their unequal access to inputs, technologies, and decision-making authority. Additionally, both nature (such as inclination towards competition or propensity for risk-taking) and nurture (including cultural and environmental influences) contribute to this distinction, as men tend to exhibit higher levels of competitiveness, lower risk-aversion, and thus, a greater inclination towards market orientation compared to women. Analyzing preferences of both men and women regarding multiple SWC attributes could guide the formulation of livelihood strategies. These strategies aim to mitigate suboptimal land use and enhance agricultural efficiency by investing in management and conservation of soil and water resources [12,7].

SWC measures encompasses enhanced farm management practices for using, placement, and extraction of resources, significantly influencing land tenure arrangements [13]. Guaranteed land rights and conservation-oriented land use offer advantages and motivations that encourage investments in SWC. Hence, the characteristics of soil and water play a pivotal role in overcoming land limitations and fostering agricultural development to ensure food security. SWC attributes such as farm consolidation and farming systems are crucial for the sustainable intensification of production [14]. Physical and structural SWC measures play an essential role in sustainable land management, proving effective in enhancing productivity and enhancing livelihood of smallholder farmers [15]. Implementing SWC practices to diversify livelihood is a key strategy to enhance resilience among smallholders against shocks [16]. Nevertheless, ineffective implementation and inadequate adoption of SWC practices, coupled with limited livelihood opportunities in off-farm sector, lead to decreased soil nutrients, reduced agricultural productivity, and adverse impacts on hydrology and environmental sustainability [17]. Therefore, SWC strategies have the potential to empower household members to make decisions about their engagement in farm and off-farm activities, as well as the allocation of revenues from these various livelihood sources [13]. It was argued that gender-specific preferences influence decision-making concerning land tenure security and the extent of family members’ involvement in adopting SWC practices [18].

In this study, we explore gender differences by analyzing smallholders' preferences for multiple attributes of SWC as integral components of sustainable agricultural intensification practices. The analysis informs the development of production and soil and water conservation strategies designed to mitigate gender inequalities in agricultural production and enhance natural resources management. Previous studies have extensively examined gender in the adoption of SWC technologies and the broader impact of gender on farm production and soil conservation [19,18]. Additionally, a substantial body of literature exists on the determinants of SWC technologies and their effects on enhancing farm productivity [[20], [21], [22], [23]]. Other studies based on sex-disaggregated data, typically centered on the household head, to investigate gender disparities in the adoption of SWC practices [24,25]. However, relying solely on such an information may overlook the contributions of other household members, including youth, in decision-making processes, thereby limiting their involvement in adopting SWC practices. Previously, studies have broadened the scope beyond household headship to evaluate gender-specific adoption decisions in plots managed jointly by males and females. These studies have produced varied results regarding whether technology adoption decisions are made collectively or individually, highlighting the importance of understanding the preferences of all household's members as a precursor to comprehending integrated household-level adoption of sustainable intensification practices in northern Rwanda [26,27]. Therefore, formulating policies solely based on the preferences of the household head may lead to misguided outcome, as decisions regarding SWC adoption are influenced by households' circumstances, social and cultural norms, and disparities in access to inputs and information between male and female members of the household [8,28].

Despite the increasing adoption of multiple SWC technologies, there remains a dearth of empirical data that specifically outlines the distinct preferences of males and females regarding SWC. This limitation impedes the development of strategies aimed at reducing gender disparities in agricultural production, conservation of soil and water resources, and livelihood diversification among rural housheolds. As noted by Ref. [29], this situation is compounded by various factors including behavioral aspects such as risk and ambiguity preferences, constraints related to access to information and credit, insecure land tenure arrangements, and inconsistent availability of necessary inputs. As emphasized by Ref. [30], research employing experimental and theory-based risk preference methods, demonstrates substantial divergences in preferences between males and females that influence their decisions regarding farm production, and water conservation practices. Therefore, overlooking gender-based preferences when formulating effective policies to promote sustainable intensification could lead to the slow adoption of SWC practices and potentially introduce biases in understanding the factors crucial for enhancing and developing sustainable intensification programs in the study area.

This study aims to evaluate gender preferences for multiple SWC practices, contributing to the methodology of the best-worst scaling (BWS) experiment for analyzing gender preferences for various attributes of SWC in Rwanda. Unlike choice experiments (CE) and contingent valuation (CV), which are the most used stated preference methods, BWS provides additional information about individual preference by allowing respondents to indicate their most or least preferred attributes across multiple SWC attribute levels. This study also provides rich knowledge on heterogeneity, complementarity or synergies, and substitutable SWC attributes that are important for policymakers to design more efficient production, conservation, and livelihood strategies.

This study is organized as follows. The next section outlines the methodology, including details on the study area, data collection procedure, the design of BWS experiment, model specification of BWS, and data analysis. Section three discusses the findings of the study. Finally, section four presents the conclusion and policy implications.

## 2 Methodology

### 2.1 Northwest volcanic agro-ecological zone study area

**Fig. 1:**
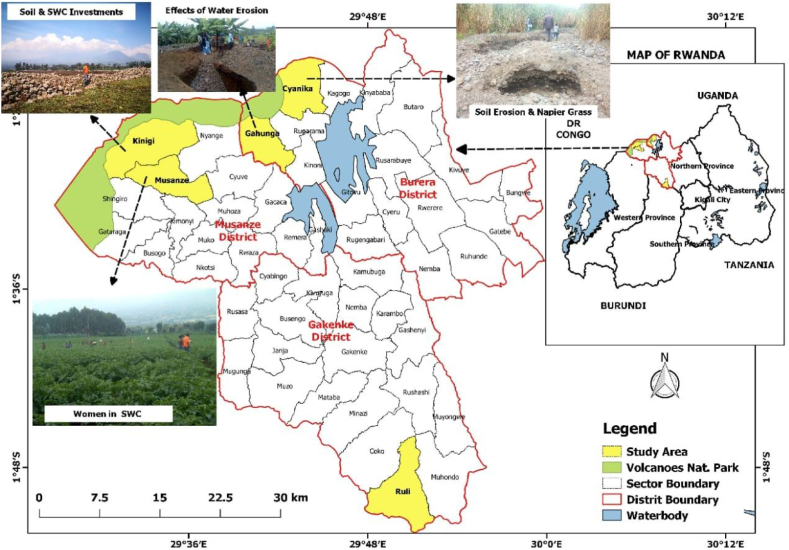
Map of the study area.

The area where the study was conducted is the northern province of Rwanda, covering three districts: Burera, Gakenke, and Musanze (see Fig. 1). The districts of Burera and Musanze are located in the northwest volcanic agro-ecological zone, with geographic coordinates of 1° 25′ S and 29° 44′ E for Burera, and 1° 29′ S and 29° 38′ E for Musanze. This area shares a border with the Republic of Democratic of Congo (DRC) and Uganda, specifically falling within the Southwest potato, sorghum, and vegetable zone. Gakenke district extends into the East Congo–Nile Highlands, with geographic coordinates of 1° 69′ S and 29° 26′ E.

The northwest volcanic agro-ecological region boasts favorable conditions for producing NTAE crops extensively. Potato production in the area currently ranges from 12 MT/ha to 25 MT/ha. Due to its high production potential in NTAE crops, the region serves as a marketing hub in East and Central African region [31]. Additionally, this region contributes 54 % to the national maize output. The adoption rate of climbing beans in the area is nearly 100 %, with annual yields surpassing 330,000 MT and productivity standing at 1.8 MT/ha.

The production system in the region heavily relies on small and fragmented land holdings, exacerbated by frequent heavy rainfall that leads to erosion and necessitates substantial investment in SWC measures. For instance, the area experiences frequent heavy rains accompanied by stones, which in recent years have significantly increased erosion risks and contributed to the formation of severe gullies near the Volcanoes National Park (VNP). Additionally, the ongoing conversion of land for agricultural purposes has accelerated soil erosion rates. As consequences, the region has witnessed a notable decrease in per capita agricultural land availability per households, dropping from 3 ha to less than one. More than 80 % of the population engages in small-scale agriculture, female labor predominates in agricultural production and soil and water conservation activities, contributing to a noticeable gender gap in livelihoods.

### 2.2 Study design

The study employed a multistage sampling technique to collect quantitative data for the BWS survey on multiple SWC from October to December 2019. Enumerators were recruited and trained to administer the BWS survey through face-to-face interviews. In the initial stage, Burera, Musanze, and Gakenke were selected based on their significant production of NTAEs crops under consideration through the feminization, agricultural transition and rural employment (FATE) project. The second stage involved proportionate sampling of five administrative sectors, including two in Burera, two in Musanze, and one in Gakenke. In the third stage, two administrative cells were randomly selected within each sector, and within each cell, two villages were chosen. For the fourth stage, the study used a systematic random sampling to select male and female respondents, selecting every fourth household from the sampling frame. The total sample consists of 653 respondents, comprising 253 males and 400 females from 422 households.

### 2.3 Best–worst scaling experiment and multiple SWC attributes

This section explores the selection and measurement of multiple SWC attributes. The study employed a BWS experimental survey to analyze preferences for attribute-levels of SWC among males and females. Compared to other valuation methods, BWS offer ex-ante insights into different approaches for eliciting farmers' choices to adopt SWC practices [32]. In this study, the selection and categorization of attribute – levels (presented in Table 1) were informed by a comprehensive literature review on agricultural investments and SWC programs, considering their policy relevance in the Rwandan context. After reviewing and selecting multiple SWC attributes, insights were gathered through focus group discussions (FGDs) and key informants’ interviews (KIIs) to enrich and refined these SWC attributes. Additionally, there were consultations with key stakeholders in agricultural production and conservation of natural resources using KIIs. Discussions sessions were also held with beneficiaries through FGDs to identify, refine, and rank attribute levels based on the perceived importance or necessity for improvement from the current status (base attribute level or status quo). Each subsequent attribute level within the same category was regarded as an enhancement over the previous one. A designed experiment was conducted to pretest SWC attributes, assessing their suitability and adaptability within local contexts. Ultimately, the study identified seven SWC attributes and 17 corresponding levels.

**Table 1:** SWC attributes and their levels.

SWC attributes	Attribute levels	Notation
Land consolidation	No-land consolidation	NLC
	Yes-land consolidation	YLC
Physical and structural measures	Grassed ridge farming	SGRFA
	Hedgerows and agroforestry	SSHAG
	Waterways and anti-erosion measures	SWATED
Soil fertility management practices	Organic and chemical fertilizer	FMOC
	Fertilizer and improved seeds	FMEP
	Fertilizer, improved seeds and water management	FMEPWA
	Management of irrigation water	FMWUA
Intra-household decisions regarding SWC	Sole female decisions in SWC	HSDM
	Joint male–female decisions in SWC	HJDM
	Integrated youth decisions in SWC	HYOUDM
SWC for diversified livelihoods	On- farm activities	LOAFS
	Off-farm activities	LOFFAS
Integrated SWC within land tenure arrangements	Existing Land tenure arrangements	LCUTE
	New land tenure arrangements	LITE

The land consolidation attribute was categorized into two levels: participation or non-participation in land consolidation, essential for promoting sustainability under the crop intensification programs in Rwanda. Sustainable crop intensification contributes to economic development and poverty reduction, meeting national food demand amidst rapid population growth. Furthermore, it enhances profitability, facilitates market and economic integration, and improves overall household well-being [33,34,35].

Attributes associated with physical and structural measures were defined in three attribute levels, aimed at directing the protection, conservation, and management of soil and water resources. Conversely, the attribute of soil fertility management (SFM) was categorized into four levels: organic and chemical fertilizer use; fertilizer combined with improved seeds; fertilizer, improved seeds, and water management; and irrigation water resources management. These levels aim to enhance soil fertility through integrated practices involving fertilizers, seeds and water management on farms. When integrated into the local knowledge and household decision-making, either individually or in combination, these attributes can signify improvements from baseline conditions. The attribute level involving the management of irrigation water through water users’ associations (WUAs) is crucial for collective action in safeguarding and managing SFM practices on farms. Key functions of WUAs include water allocation and distribution, collection of service fees, and maintenance of SWC infrastructure [36].

The attribute of intra-household decisions regarding SWC reflects the perceptions regarding the control exerted by men, women, and youth over practical decisions, as well as their ability to invest in SWC strategies, which are important optimization strategies [37]. Another key attribute to consider is the SWC for diversified livelihoods, which assesses how SWC structures influence the income and productivity of smallholder farmers [38]. SWC measures are crucial in improving the livelihoods of rural farm households through on-farm and diversification in off-farm activities. Attributes involving “integrated SWC in land tenure arrangement” attempts to answer the question of whether households’ ownership of plots (whether owned, rented out, or rented in) can improve investment in SWC [39]. explored the significance of tenure security in shaping the extent of SWC adoption and proposed the necessity for policies aimed at enhancing tenure security to foster enduring SWC initiatives.

The study used a BWS experiment to determine the differences in gender preferences for the SWC strategies. BWS is rooted in the theoretical framework outlined by Ref. [40]. This method involves respondents in ordering tasks where they indicate their preferred options. Survey participants are prompted to select the most important and least important items from two or more alternatives, with each SWC attribute level varies across these alternatives [41].

BWS manifests in three distinct cases: the BWS object case (involving the selection of attributes as best and worst), the BWS profile case (where attribute levels are chosen as best and worst), and the BWS multi profile case, which entails selecting profiles as best worst [42,43]. This study utilized the BWS multiprofile case, where respondents were tasked with selecting among multiple SWC attributes and their levels in a choice set. This approach represents a more recent extension of choice experiment (CE), addressing limitations observed in the two BWS cases. It offers richer insights by leveraging respondents’ tendencies to identify extreme preferences and is adaptable for individual-level analysis. BWS methods have gained extensive application in health economics [44,45]. In the agricultural domain, BWS and latent class cluster analysis were employed to explore agriculture marketing channels and assess consumer preferences regarding pork attributes [46]. The application of BWS in Rwanda is particularly innovative, specifically in assessing gender preferences for agricultural investment within a developing country context.

### 2.4 Best–worst scaling experimental set up and design

**Fig. 2:**
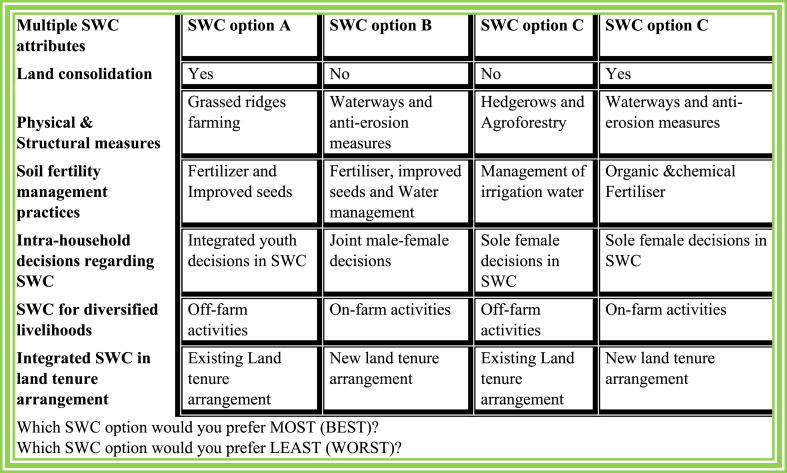
A sample of multiple SWC choice card.

The BWS experimental design consists of combining seven SWC attributes (K=7) and their levels (L=17). SWC attributes used in the design had two, three, or four levels (L=2,3,4). These BWS attribute levels can be employed to model individual-level choices involving six to ten attributes, with each attribute having levels ranging from two to four. This indicates that the designed BWS attributes and levels was viable and could give reliable choice sets. An example of one of the samples of a multiple SWC choice card is illustrated in Fig. 2. Using a complete factorial design that involves an entire attribute-level combination with an LK number of scenarios could yield 1152 maximum best–worst choices. However, the above full factorial design is not feasible for practical analysis. A fractional factorial approach involving the orthogonal design can provide optimal estimates.

In this study, an orthogonal main-effect design plan (OMEPs) was adopted using IBM SPSS statistics to generate SWC choice sets. OMEP proved to be a practical choice for multiple SWC attribute levels, including scenarios with uneven-level replication across more than one attribute [47]. The orthogonal design resulted in a total of 64 combinations of SWC attribute levels. Unlike CE, which generates paired best–worst choices and excludes one alternative as the status quo, our approach involved grouping into every best–worst-choice set. Respondents evaluated each SWC option presented on choice cards, identifying their most preferred and least preferred options; and selecting their preferred option from the remaining two SWC options. In summary, all 64 combinations were grouped into eight profiles and 16 best–worst choice sets, each containing four alternatives or options.

### 2.5 Model specification and data analysis

Research on gender preferences extends from the theoretical groundwork of the best–worst scaling experiment provided by Ref. [40] and is grounded in random utility theory (RUT) by McFadden (1974). According to the RUT, decision-makers optimize their utility by selecting their preferred bundle from a set of SWC options. The application of RUT to BWS was elucidated using the maximum difference (maxdiff) model in Equation (1). The potential choices of BWS across multiple levels of SWC attributes were established as pairs of best and worst selections. In the estimation process using multinomial logit (MNL) with maximum difference, a dual coding was employed, such that best = 1 and best = 0. Hence, best was coded as 1 if a farmer chose the most important SWC attribute level as their top priority, and the best equals 0 otherwise. Similarly, worst was coded as 1 if a farmer identified the least important SWC attribute level as their least preferred choice, and worst equals 0 otherwise [45]. It was assumed the error term follow a Gumbel distribution for each pair of best–worst-choices.

(1) $$P_{BW}\left(\frac{ii^{\prime }}{X}\right)=\frac{\exp\left(\mu \left(\sum_{k=1}^{n}(\alpha_{i}X_{ki}-\alpha_{i}X_{ki^{\prime }}\right)\right)}{\sum_{j^{\prime \neq }j}j,j^{\prime }}\in Mexp(\mu \left(\sum_{k=1}^{n}(\alpha_{j}X_{kj}-\alpha_{j}X_{ki^{\prime }}\right)$$

where $X_{ki}$ denotes the attribute level of SWC chosen as the potential best option in the profile, $X_{{ki}^{\prime }}$ represents the SWC attribute level chosen as the potential worst option, and μ is the parameter governing the scale of utilities. Parameter vectors $\alpha_{i}$ corresponds to $X_{ki}$, while $\alpha_{i^{\prime }}$ corresponds to $X_{{ki}^{\prime }}$.

For econometric analysis, this study used the maximum difference (maxdiff) with MNL model to examine how respondents express preferences across multiple levels of SWC attribute, particularly focusing on their best and worst choices. The likelihood of selecting a specific pair within a BWS choice set, which maximizes the difference between the “worst” and the “best” SWC attribute level, is directly related to the perceived difference in their importance [48]. Equation (1) illustrates this relationship, and the maxdiff with MNL-based model appears in Equation (2). In the context of MNL, it is assumed that the utility derived from selecting the most important option is exactly opposite in sign to the utility derived from selecting the least important option [48].

(2) $$U_{{Male}_{diff}^{i}}^{{Female}_{diff}^{i}}=\beta_{NLC}D_{NLC}^{i}+\beta_{SGRFA}D_{SGRFA}^{i}+\beta_{SSHAG}D_{SSHAG}^{i}+\beta_{SWATED}D_{SWATED}^{i}+\beta_{FMOC}D_{FMOC}^{i}+\beta_{FMOP}D_{FMOP}^{i}+\beta_{FMEPWA}D_{FMEPWA}^{i}+\beta_{FMWUA}D_{FMWUA}^{i}+\beta_{HSDM}D_{HSDM}^{i}\;+\beta_{HJDM}D_{HJDM}^{i}+\beta_{HYOUDM}D_{HYOUDM}^{i}+\beta_{LOAFS}D_{LOAFS}^{i}+\beta_{LOFFAS}D_{LOFFAS}^{i}+\beta_{LCUTE}D_{LCUTE}^{i}+\beta_{LITE}D_{LITE}^{i}$$

For option i, the SWC attribute level considered most important ($D_{swc\;attribute-level}^{i}$) was assigned a value of 1, whereas the least important attribute level was assigned −1; the third attribute level was set to 0.

To assess individual preferences, both separately for males and females and combined, using the MNL model, land consolidation (YLC) was selected as the reference SWC attribute level and treated as the omitted variable with a utility scale value set to 0 during both model specification and regression analysis. Following this, the utility value of YLC was subtracted from all other attribute levels to determine their relative importance. Coefficients for each attribute level were estimated relative to land consolidation, and preferences for the rest of attribute levels were evaluated based on their magnitude and direction relative to land consolidation (YLC) [49]. The choice of land consolidation (YLC) attribute level emphasizes the critical need to promote sustainable crop intensification and facilitate market integration in response to the increasing land scarcity to the high requirements in food due to the growing population in the study area [33,34].

The study used various methods, including counting scores (disaggregated by sex of the respondent), relative attribute-level importance, and probability ratio to characterize multiple SWC attribute levels. From the mean B–W scores were used in the relative importance approach to assess the significance of each SWC attribute level for all male and female farmers with reference to sustainable integration. The relative importance values were interpreted as probabilities indicating the likelihood of farmers selecting a particular SWC attribute level as the most important among several options [50]. In analyzing the relative importance, the study highlighted the paramount importance of the attribute level pertaining to the “integrated youth decisions in SWC”, assigning it the highest index scale to 100 % on an interval scale and adjusted accordingly. Subsequently, probability ratio scale methods were used to calculate the averages of all B–W coefficients, underscoring their relationships with the variance–covariance matrix.

To capture both the heterogeneity of attribute-level importance (variance) and the correlation among attribute levels (covariance) in this study, we computed the variance-covariance matrix using individual B–W scores. For each SWC attribute level across respondents, variance-covariance values were derived from all the means and standard deviations of individual B–W scores. The standard deviation across the respondents measured the extent of variation in the relative importance of SWC attribute levels within the sample. Higher standard deviations indicated greater disparity among respondents. Hence, variances or standard deviations were used to assess the degree of heterogeneity.

## 3 Results and discussion

### 3.1 Analysis of count scores of multiple SWC attribute levels

Table 2 presents the ranking of multiple SWC attribute levels based on utility scores of best and worst. The results reveal that both male and female farmers identified several SWC attribute levels with the highest scores as the most important attributes. These include “on-farm activities” (1487), waterways and anti-erosion measures, integration of youth decisions in SWC (1,462), no-land consolidation (1,389), and improved tenure (1,324).

**Table 2:** Count analysis of multiple SWC attributes.

SWC attributes		Best scores	Worst scores	Aggregated (B–W)	Mean of (B–W)	St. Dev (B–W)	Sqrt B–W	Sqrt stand.
Land consolidation	No-land consolidation	1389	1291	98	−0.1	1.39	4.7	67.8
	Land consolidation	1197	1231	−34	0.2	1.52	3.5	49.4
Physical and structural measures	Grassed ridge farming	667	607	60	0.1	1.44	2.6	37.0
	Hedgerows and agroforestry	838	887	−49	−0.1	1.13	5.1	72.5
	Waterways and anti-erosion measures	1485	926	155	0.3	1.67	2.9	42.2
Soil fertility management practices	Organic and chemical fertilizer	629	571	58	0.1	1.20	4.1	59.2
	Fertilizer and improved seeds	460	592	−132	−0.2	1.33	1.4	20.3
	Fertilizer, seeds, and water management	475	384	91	0.2	0.97	2.7	38.9
	Management of irrigation water	758	895	−137	−0.2	1.32	4.1	59.2
Intra-household decisions regarding SWC	Sole female decisions in SWC	570	345	−304	−0.5	1.48	2.7	38.9
	Joint male–female decisions in SWC	980	629	635	1.0	1.45	4.5	65.1
	Integrated youth decisions in SWC	1462	874	833	1.3	1.86	7.0	100.0
SWC for diversified livelihood	On-farm activities	1487	1137	350	0.6	1.61	3.7	53.5
	Off-farm activities	1249	1249	−18	−0.03	1.49	4.0	56.9
Integrated SWC in land tenure arrangement	Existing land tenure arrangement	1274	1137	137	0.2	1.32	1.6	22.6
	New land tenure arrangement	1324	1182	142	0.2	1.36	3.9	56.4

SWC attribute levels such as off-farm activities (1,249), land consolidation (1,231), and new land tenure arrangements (1,182) are considered moderately important, each scoring above 1000. In contrast, attribute levels scoring below 1000 are deemed less important for smallholder farmers. Given the significance of youth integration in SWC decisions and on-farm activities, combining participatory decision-making in farming could lead to the best strategy for SWC investment. The results with count analysis indicate that farmers assigned the highest scores to SWC attribute levels that integrate SWC in land tenure arrangements, participatory households’ decisions, intensification and diversified livelihoods through on-farm or off-farm employment. The preferences for higher scores for on-farm activities than off-farming suggests that smallholders prioritize working on their farms rather than diversifying their incomes through off-farm activities. This trend may be linked to an increase in rural female decision-makers within households, who play crucial roles in farm operations aimed at achieving sustainable agricultural intensification.

The results indicate that non-consolidation (1,291) holds greater importance compared to land consolidation (1,197), suggesting that diversifying into off-farm activities is prioritized over managing on-farm operations. This preference implies that farmers who perceive land consolidation as less important may not fully capitalize on the benefits provided by government programs aimed at promoting crop intensification. These initiatives include enhanced access to agricultural inputs such as seeds and fertilizers, improvement in land productivity, extension services, support for postharvest handling and storage, and enhanced water management through advanced irrigation techniques. As highlighted by Ref. [51], there exists a price disparity between consolidated and nonconsolidated zones, illustrating how programs such as CIP have increased productivity and farm incomes by optimizing land-use efficiency and influencing market prices.

Under physical and structural measures attributes, waterways and anti-erosion measures are more important SWC attribute levels than hedgerows, agroforestry, and grassed ridge farming. This suggests that smallholders’ awareness of the impact of soil and water erosion underscores the necessity for farmers to enhance the existing and commonly practiced grassed ridge farming as a SWC practice. Introducing waterways and anti-erosion measures, as well as agroforestry technologies could be instrumental in addressing these concerns [52]. demonstrated that integrating multiple SWC practices can increase crop yields, provide forage options, and enhance livestock performance. Furthermore, implementing waterways and anti-erosion measures can effectively decrease water run-off volume and velocity, diverting water towards larger water streams.

Within the household's decision-making attribute, SWC attribute level called “integrated youth decisions in SWC” was the most frequently chosen, followed by joint male–female and sole female decisions. Higher scores for youth inclusion indicate a shift in farmers' perceptions away from the traditional household decision-making process, which often reinforces labor allocation inequalities and overlook the unpaid and often invisible contributions of women and youth to agricultural labor. As argued by Ref. [53], integrating young people into land ownership and participatory decision-making could mitigate information asymmetries and bargaining power imbalances within smallholder farming households.

In the context of integrated SWC within land tenure arrangements, new land tenure arrangement holds greater significance over existing ones. This indicates that facilitates access to land for young women and men, and involving them in land-use decision-making, could spur innovation and creativity among farmers managing their own farms. Enhancing land tenure security, while increasing youth and women's participation in land consolidation efforts, has the potential to foster the development of novel, environmentally sustainable, and highly productive farming practices (White, 2015).

In terms of SFM attributes, the highest scores were achieved in the SWC attribute levels related to “management of irrigation water”. Following closely were scores related to organic and chemical fertilizer use, as well as the combined application of fertilizer, improved seeds and water management. The strong emphasis placed by respondents on participating in water management through WUAs underscores their commitment to effectively managing water resources for sustainable crop production.

Additionally, the elevated scores in both fertilizer and organic manure highlight farmers’ awareness of organic farming practices, critical for ensuring food safety. However, the attribute related to 'fertilizer and improved seeds and management of irrigation water showed a preference for worst scores over best scores, highlighting the need to raise awareness about the benefits of integrating irrigation and nutrient management strategies. As emphasized by Ref. [54], integrating climate-smart strategies into these practices is essential for sustainable crop production systems. These findings also suggest that some farmers may have misperceptions regarding the availability of improved seeds and fertilizer subsidies, particularly among those who are not actively involved in the government program known as CIP.

### 3.2 Ranking the relative importance of multiple SWC strategies by gender

The results in Table 3 illustrate the relative importance of various SWC attribute levels for the overall sample. Notably, youth integration in SWC decisions stands out with a relative importance of 100 % for both males and females. Intra-household decisions regarding SWC, diversified livelihoods in on-farming activities, and integrated SWC within land tenure arrangements were consistently ranked as the most selected and most important attribute levels by both women and men respondents. Respondents also highlighted fertilizer, pesticides, and water (92) as moderately selected but important attributes, underscoring their importance in sustainable crop production practices; whereas no-land consolidation (53) received less emphasis but was still noted as significant.

**Table 3:** Ranking multiple SWC attributes relative to their importance.

Attributes	Female relative importance (N = 400)						Male relative importance (n = 253)					
	BW scores	Av (BW)	Ranking Av. (BW)	Sqrt (BW)	R. importance (%)	Ranking	BW scores	Av. (BW)	Ranking Av. (BW)	Sqrt (BW)	R. importance (%)	Ranking
No-consolidation	53.0	0.04	8	1.0	26.8	11	45.0	0.03	10	2.4	75.4	3
Consolidation	−21.0	−0.02	11	3.1	84.3	2	−13.0	−0.01	13	1.6	48.7	11
Grassed ridge farming	19.0	0.03	10	1.0	26.8	11	41.0	0.1	4	1.6	48.7	11
Hedgerows and agroforestry	−47.0	−0.1	13	2.6	70.7	3	−2.0	−0.003	12	2.4	74.6	4
Waterways and anti-erosion measures	130.0	0.2	3	0.7	18.9	14	25.0	0.04	9	2.2	68.9	5
Organic and chemical fertilizer	25.0	0.0	9	2.1	56.8	6	33.0	0.1	5	2.0	61.8	8
Fertilizer and improved seeds	−103.0	−0.2	16	0.7	18.9	14	−29.0	−0.01	14	0.7	21.9	15
Fertilizer, seeds, and water management	62.0	0.1	5	1.0	26.8	11	29.0	0.0	6	1.7	52.8	10
Management of irrigation water	−82.0	−0.1	15	2.0	53.6	7	−55.0	−0.1	15	2.1	65.6	6
Sole female decisions in SWC	−149.0	−0.1	14	2.0	53.6	7	−155.0	−0.1	16	0.7	21.9	15
Joint male–female decisions in SWC	428.0	0.7	2	2.4	64.7	5	207.0	0.3	2	2.1	65.6	6
Integrated youth decisions in SWC	556.0	0.9	1	3.7	100.0	1	277.0	0.4	1	3.2	100.0	1
On-farm activities	220.0	0.2	4	2.4	65.4	4	130.0	0.1	3	1.3	39.7	13
Off-farm activities	−21.0	−0.01	11	1.4	37.9	10	3.0	0.0	11	2.5	78.8	2
Existing land tenure arrangement	85.0	0.1	7	0.7	18.9	14	52.0	0.0	7	0.9	26.8	14
New land tenure arrangement	91.0	0.1	6	1.9	51.7	9	51.0	0.0	8	2.0	61.8	8

Female respondents attributed notably high relative importance of 65 % and 85 % to various attribute levels compared to youth integration in SWC decisions. Notably, these attribute levels included consolidation (84.3 %), hedgerows and agroforestry (70.7 %), and on-farm activities (65.4 %). In contrast, male respondents emphasized attribute levels such as off-farming activities (78.8 %), no-consolidation (75.4 %), hedgerows and agroforestry (74.6 %), and waterways and anti-erosion measures (68.9 %) as equally crucial as the involving youth in SWC decisions.

In the highly important scenario, multiple SWC attribute levels emerge with importance ranging from 0.72 to 0.65 times (at least three-quarters) compared to the “integration of youth in SWC decisions” (considered 100 % important). Notably, key attribute levels for females include consolidation (84.3 %), hedgerows and agroforestry (70.7 %), on-farm activities (65.4 %), and joint male–female decisions (64.7 %). This scenario underscores the potential for integrating youth decisions in SWC, thereby enhancing women's involvement in land consolidation for agricultural production within their own households. This transformative process suggests that land consolidation reshapes household dynamics and empower women to engage in agricultural innovations. This justifies the increasing proportion of rural women and female household heads assuming the roles of farm managers and agricultural wage laborers.

For males, the most important scenarios integrating multiple SWC attribute levels for males consists of off-farming activities (78.8 %), no-land consolidation (75.4 %), hedgerows and agroforestry (74.6 %), waterways and anti-erosion measures (68.9 %), participation in management of irrigation water through WUAs (65.6 %), and joint male–female (65.6 %). In this scenario, the SWC attribute levels, when considered most important alongside the inclusion of youth decisions, support increased male involvement in off-farm wage work, while maintaining their roles and in managing farm resources. However, according to Ref. [55], prolonged males' absence due to labor outmigration results in married women assuming farm management roles and becoming primary contributors to agricultural production. This dynamic implies that men's absence may limit their participation in physical and structural conservation efforts, possibly because they rely on hired farm labor while working off-farm.

Using choice probabilities, the findings reveal gender disparities in preferences across various combinations of SWC attribute levels. Female farmers exhibit notably strong preferences, with relative importance scores exceeding 65 %, for integration of youth decisions in SWC, land consolidation, hedgerows and agroforestry, and on-farm activities. Similarly, male farmers demonstrate robust preferences for youth integration in SWC, off-farm activities, no-consolidation, hedgerows and agroforestry, and waterways and anti-erosion measures. The results underscore the importance of household decision-making in shaping preferences and linking farm investment technologies, land consolidation, and market participation. Farmers who invest in these strategies can maximize production and contribute significantly to environmental sustainability.

The results reveal a scenario where SWC attribute levels are moderately important, valued at least half (>0.5) as much as the most important attribute level, “integration of youth decisions in SWC”. For females, the attribute levels include joint male–female decisions, organic and chemical fertilizer use, participation in managing irrigation water through WUAs, and new land tenure arrangements (>0.5). This underscores females’ prioritization of integrated land and household decisions as important aspects of farm management and collective action. Conversely, for males, this scenario comprises attribute levels such as new land tenure arrangements, fertilizer, improved seeds and water management, grassed ridge farming, and land consolidation. This suggests that men prioritize enhanced land and household decisions, potentially driving farmers participating in consolidation programs to optimize the use of farm resources efficiently.

The rest of the attribute levels form the basic scenario of SWC. The least preferred scenario comprises attribute levels with a relative importance below 50 %, implying that they are less than half as important as the most critical attribute scenarios. Among females, attribute levels with notably low importance are existing land tenure arrangements, grassed ridge farming, fertilizer, improved seeds and water management, no-consolidation, and off-farm activities. Similarly, for male, low importance was placed on existing land tenure arrangements, on-farm activities, SWC decisions solely by females, and fertilizer and improved seeds. The scenario underscores a shift towards recognizing distinct preferences between males and females, diverging from traditional gender roles in land management where men predominantly dictated land management and household decisions, while women were predominantly engaged in household chores and primary production activities.

### 3.3 Heterogeneity of SWC attribute-level importance

Table 4 presents the significance of B–W measures, such as mean (B–W) and standardized Sqrt (B–W). The results on heterogeneity looks similar with the results of the ranking methods of importance discussed previously. For instance, the intermediate mean score of attribute levels such as improved tenure, participation in management of irrigation water through (WUAs), and fertilizer organic and chemical use indicates that all females perceived these attribute levels as moderately important on average. Similarly, males rate attributes such as fertilizer, improved seeds, and water management; organic and chemical fertilizer use, and new land tenure arrangements as moderately important. This suggests that averaging out responses reflect a blend of opinion from both females or males, where some consider these attribute levels highly important while others find them less significant [50].

**Table 4:** Multiple SWC attribute-level importance and individual SWC scores.

Attributes	Female (n = 400)							Male (n = 253)						
	B	W	Agg. (BW)	Mean (BW)	St. Dev (BW)	Sqrt B/W	Sqrt st.	B	W	Ag. (BW)	Mean (BW)	St. Dev (BW)	Sqrt B/W	Sqrt st.
No-land consolidation	840	787	53	−0.05	1.40	3.15	84.3	549	504	45	−0.05	1.39	1.58	48.7
Yes-land consolidation	734	755	−21	0.13	1.53	1.00	26.8	463	476	−13	0.18	1.51	2.44	75.4
Grassed ridge farming	392	373	19	0.05	1.44	1.00	26.8	275	234	41	0.16	1.46	1.58	48.7
Hedgerows and agroforestry	509	556	−47	−0.12	1.14	2.64	70.7	329	331	−2	−0.01	1.11	2.41	74.6
Waterways and anti-erosion measures	918	547	130	0.33	1.66	0.71	18.9	567	379	25	0.10	1.66	2.23	69.0
Organic and chemical fertilizer	380	355	25.0	0.06	1.23	2.12	56.8	249	216	33	0.13	1.16	2.00	61.8
Fertilizer and improved seeds	264	367	−103	−0.26	1.30	0.71	18.9	196	225	−29	−0.11	1.37	0.71	21.9
Fertilizer, seeds, and water management	288	226	62.0	0.16	0.94	1.00	26.8	187	158	29	0.11	1.01	1.71	52.8
Management of irrigation water	460	542	−82	−0.21	1.30	2.00	53.6	298	353	−55	−0.22	1.36	2.12	65.6
Sole female decisions in SWC	353	184	−149	−0.37	1.47	2.00	53.6	217	161	−155	−0.61	1.49	0.71	21.9
Joint male–female decisions in SWC	612	373	428	1.07	1.43	2.41	64.7	368		207	0.82	1.46	2.12	65.6
Integrated youth decisions in SWC	929	502	556	1.39	1.83	3.73	100	533	372	277	1.09	1.90	3.24	100
On-farm activities	904	684	220	0.55	1.57	2.44	65.4	583	453	130	0.51	1.68	1.28	39.7
Off-farm activities	773	773	−21	−0.05	1.45	1.41	37.9	476	476	3	0.01	1.55	2.55	78.8
Existing land tenure arrangement	772	687	85	0.21	1.26	0.71	18.9	502	450	52	0.21	1.42	0.87	26.8
New land tenure arrangement	814	723	91	0.23	1.31	1.93	51.7	510	459	51	0.20	1.45	2.00	61.8

The standard deviation for male and female B–W scores shows that, except for fertilizer, pesticide, and water management, other SWC attribute levels have scores above one. This indicates significant heterogeneity in how these SWC attribute levels are perceived across respondents (both male and female). Scores below one signal uniform perceptions and nearly identical adoption decisions concerning integrated use of fertilizer, improved seeds, and water management in the area. The high variance and standard deviation of individual B–W scores imply that perception and adoption decisions vary widely across different attribute levels.

This variability across attributes underscores the diverse importance placed on each attribute level by the respondents and measures the degree of heterogeneity in adopting multiple SWC practices [50]. The results reveal gender differences in the relative importance of attribute levels, particularly land consolidation, waterways and anti-erosion measures, integration of youth in SWC decisions, and on-farming activities. Females exhibit higher agreement and lower heterogeneity regarding fertilizer, improved seeds, and water management compared to men.

Table 5 presents the correlation results between attribute levels for both male and female farmers in the study area. A positive correlation indicates synergies or complementarities between two attribute levels. For instance, the following attribute levels are positively correlated: non-consolidation and fertilizer/improved seeds (0.31), integration of youth decisions in SWC and joint male–female decisions (0.34), and new land tenure arrangements and fertilizer/improved seeds (0.36). Conversely, moderate to high negative association suggests trade-off between various SWC attribute levels. Negative correlations were observed between joint male–female decisions and organic/chemical fertilizer (0.36), joint male–female decisions and sole female decisions (0.37), integration of youth decisions in SWC and fertilizer/improved seeds (0.40), waterways and anti-erosion measures and grassed ridge farming (0.54), non-consolidation and consolidation (0.62), and on-farm and off-farming activities (0.88). The results with the Pearson correlation matrix reveal that most attribute levels have an extremely low significant correlation, which is 0.3 (r < 0.3). There is a low correlation since the coefficients are r < 0.35, and a moderate to high correlation since r > 45.

**Table 5:** Pearson correlation matrix of multiple SWC attributes.

Attributes	NLC	YLC	SGRFA	SSHAG	SWATED	FMOC	FMEP	FMEPWA	FMWUA	HSDM	HJDM	HYOUDM	LOAFS	LOFFAS	LCUTE	LITE
**YLC**	−0.8^a^	1.00														
**SGRFA**	0.2^a^	0.1^b^	1.0													
**SSHAG**	−0.1^a^	0.2^a^	−0.3^a^	1.0												
**SWATED**	0.1^c^	0.1^b^	0.5^a^	−0.4^a^	1.0											
**FMOC**	0.01	0.2^a^	−0.02	−0.01	0.2^a^	1.0										
**FMEP**	−0.1	0.3^a^	0.2^a^	0.1^c^	0.05	−0.2^a^	1.0									
**FMEPWA**	0.1^a^	0.1^c^	0.2^a^	−0.04	0.1^c^	0.1^a^	0.2^a^	1.0								
**FMWUA**	0.3^a^	−0.1	0.05	0.05	0.1^b^	−0.1^b^	−0.2^a^	−0.3^a^	1.0							
**HSDM**	−0.1^c^	−0.1^a^	−0.03	0.03	−0.1^a^	0.1^b^	−0.4^a^	−0.04	−0.03	1.0						
**HJSD**	−0.1^c^	−0.1^b^	0.03	−0.05	−0.05	0.2^a^	−0.1^a^	0.1^c^	−0.2^a^	0.1^a^	1.0					
**HYOUDM**	0.04	−0.1^a^	−0.1^c^	−0.05	−0.1^b^	−0.1^c^	−0.3^a^	0.1^b^	−0.2^a^	0.3^a^	0.2^a^	1.0				
**LOAFS**	0.1^a^	0.2^a^	0.3^a^	0.1^c^	0.05	−0.1	0.2^a^	0.2^a^	−0.1^c^	−0.1^b^	0.2^a^	0.02	1.0			
**LOFFAS**	−0.03	0.1^a^	−0.1^c^	0.1	0.1^a^	0.3^b^	0.1^a^	0.03	0.3^a^	0.05	−0.2^a^	−0.2^a^	−0.7^a^	1.0		
**LCUTE**	0.05	0.2^a^	0.1	−0.1^a^	0.3^a^	0.2^a^	0.3^a^	0.04	0.01	−0.02	0.03	−0.01	0.1^a^	0.2^a^	1.0	
**LITE**	0.2^a^	0.1^b^	0.2^a^	0.2^a^	0.02	−0.1	0.1^b^	0.2^a^	0.2^a^	0.04	−0.04	−0.1^c^	0.2^a^	0.1	−0.6^a^	1.0

Note levels of significance of 1 %, 5 %, and 10 % respectively for.

a , p < 0.001.

b , p < 0.01.

c , p < 0.1.

Furthermore, the correlation coefficients, predominantly different from zero, suggesting a substantial structure influenced by a clear model specification and interdependence among attribute levels. The results reveal a statistically significant, albeit modest, positive relationship between attribute levels such as non-consolidation, and fertilizer and improved seeds (0.31). This implies that preference for the lack of participation in land consolidation correlates with limited access to improved seeds and fertilizers, probably due to exclusion from the government subsidy program linked with Rwanda's CIP and land consolidation requirements. The study also identifies the modest synergetic relationship between attribute levels such as the integration of youth decisions in SWC and joint male–female decisions (0.34), largely attributed to differing perceptions of household decision-making roles between parents and young members. Hence, prioritizing youth involvement in SWC decisions may not be a priority when spouses jointly make decisions. Preferences for new land tenure arrangements are positively associated with fertilizer/improved seeds (0.36). This complementary association reveals that enhanced access to land tenure rights encourages farmers to invest in acquiring improved seeds and fertilizers for agricultural development. The low correlation indicates little competition for scarce resources among household members when adopting SWC practices.

Additionally, the results unveiled a moderately negative but significant relationship between joint male–female decisions and the use of organic and chemical fertilizer (0.36), joint male–female decisions (0.37), waterways and anti-erosion measures, grassed ridge farming (0.40), as well as the integration of youth decisions. The results support earlier conclusions that decision-making regarding the adoption of physical and structural measures, as well as SFM practices, does not require the active involvement of every household member. Results dignifies that women are assuming full managerial and labor provision roles and making decisions regarding SWC adoption. The application of waterways and anti-erosion measures showed a significant negatively and significantly associated with grassed ridge farming (0.54), possibly due to farmers’ limited knowledge or extension information on how these practices the role and complement each other on the farm. This suggests a lack of understanding regarding the role of anti-erosion measures in channeling water away from production areas and redirecting it to waterways, as well as the role of vegetation or grasses integrated with agroforestry trees in reducing soil run-off and promoting infiltration. High trade-offs were observed between non-consolidation and consolidation (0.62), as well as between on-farm and off-farming activities (0.88). These findings suggest that respondents have little interest in participating in land consolidation programs and limited capacity to engage in diversified livelihood sources.

### 3.4 MNL model for multiple SWC attribute levels

The best–worst results, obtained through the MNL model and maximum difference analysis, are presented in Table 6. Given the escalating population density, there is a pressing need for widespread promotion of efficient and sustainable agricultural practices enhance agricultural productivity, particularly in the populated northern Province [56].

**Table 6:** MNL estimation of gender preferences for multiple SWC attributes.

Attributes	Female respondents (n = 400)	Male respondents (n = 253)	Overall Respondents (n = 653)
Yes-land consolidation	**(Base outcome)**		
No-land consolidation	−6.05^a^ (0.78)	−13.29^a^ (4.14)	−2.99^a^ (0.31)
Grassed ridge farming	2.40^a^ (0.50)	5.41^a^ (2.19)	0.72^a^ (0.24)
Hedgerows and agroforestry	1.84^a^ (0.50)	3.71^2^^b^ (1.77)	0.39^c^ (0.23)
Waterways and anti-erosion measures	2.11^a^ (0.46)	4.30^b^ (1.96)	0.48^b^ (0.24)
Organic and chemical fertilizer	1.04^a^ (0.29)	3.34^b^ (1.38)	0.7^a^ (0.22)
Fertilizer and improved seeds	0.89^a^ (0.28)	2.72^a^ (1.02)	0.08 (0.28)
Fertilizer, seeds and water management	0.46^c^ (0.26)	1.10 (0.75)	−0.11 (0.23)
Management of irrigation water	0.69^a^ (0.26)	2.09^b^ (0.80)	−0.24 (0.25)
Sole female decisions in SWC	0.02 (0.15)	−0.14 (0.37)	−0.46^c^ (0.25)
Joint male–female decisions in SWC	−0.68^a^ (0.18)	−1.74^a^ (0.67)	−0.4^c^ (0.21)
Integrated youth decisions in SWC	−0.02 (0.13)	0.13 (0.36)	−0.51 (0.23)
On-farm activities	1.63^a^ (0.47)	1.56 (1.10)	0.87^a^ (0.25)
Off-farm activities	0.76^c^ (0.44)	−0.88 (1.27)	0.63^a^ (0.26)
Existing Land tenure arrangement	1.34^a^ (0.40)	2.86^b^ (1.33)	0.76^a^ (0.25)
New land tenure arrangement	1.15^a^ (0.39)	2.96^b^ (1.35)	0.73^a^ (0.25)
_cons	−4.17^b^ (2.13)	−5.72 (5.10)	−4.04^a^ (0.97)

Note: Log likelihood = −313.13886; LR chi2 (17) = 245.14; Prob > chi2 = 0.0000; and Pseudo R2 = 0.2813.

-Significance levels are indicated respectively as.

a p value < 0.001.

b p value < 0.01; and.

c p value < 0.1, suggesting 1 %, 5 %, and 10 % levels of. Standard errors are in brackets.

Overall, the log-likelihood ratio, chi-square test, and p-value collectively indicate that the fit of the MNL model was satisfactory. The value of log likelihood value is −313.13886 and chi-square test of Prob > chi2 = 0.0000 indicate that both significant at 1 % level, demonstrating a string overall fits of the MNL model.

The results show a significant negative association between attribute levels of no-land consolidation and land consolidation, which is statistically significant at 1 %. This suggests that both male and female farmers who prioritize land consolidation less likely to believe that non-consolidation is important. Men exhibit stronger preferences than women (−13.29 to −6.05) for activities associated with no-consolidation and other forms of off-farm employment rather than consolidating their lands for agricultural activities.

The results indicate that attribute levels related to SFM are statistically significant and positively correlated with land consolidation. This indicates a strong preference among both men and for these specific attribute levels over others, reflecting an increasing trend among both genders to transition farming methods from a sole focus on productivity to prioritizing sustainable production. These transition pathways aim to reshape existing farming system through farm structural changes that enhance sustainable food production [57]. This shift may stem from farmers’ knowledge of the integrating and innovatively applying improved management techniques to promote sustainable crop production.

Findings indicate that all attribute levels associated with physical and structural measures significantly contribute to land consolidation. Waterways and anti-erosion measures, and grassed ridge farming are particularly favored over hedgerows and agroforestry. Both men and women show a preference for improving current farming and conservation practices, frequently applying waterways and anti-erosion measures to conserve soil and protect water. Nevertheless, there seem to be limited awareness among both genders regarding the benefits of integrating of hedgerows and agroforestry trees on their land. Integrating all physical and structural measures of SWC has proven effective in reducing the amount, velocity, and rate of surface runoff, thereby mitigating environmental damages caused by runoff. These measures also aim to increase plant height, improve crop yield, and enhance productivity by reducing soil erosion and preserving soil vegetation and cover, and consequently boosting soil organic matter content in the farm. Moreover, these SWC measures have the potential to enhance resource use efficiency for sustainable crop production [58].

The attribute levels related to SFM, such as organic and chemical fertilizers, fertilizers combined with improved seeds, and management of irrigation water through WUAs, are statistically significant and positively associated with land consolidation among both male and female farmers. Both male and female farmers show a shared commitment to WUAs for managing water resources from nearby volcanoes, engaging in activities like digging holes, creating ditches, and constructing water channels. However, while attribute levels such as fertilizer, improved seeds, and water management are significant factors for females at10 %, they do not hold the same significance for males. The preference for fertilizer use highlights the reputation of land consolidation in promoting intensive biotechnology adoption, which facilitates farmers' access to subsidized agricultural inputs, particularly chemical and customized extension services by local extension agents [59]. Under programs such as the CIP, local extension agents play a crucial role in overseeing the distribution of subsidized inputs and providing guidance to smallholder farmers organized into cooperatives [35]. This underscores the government role in increasing farmers’ awareness of the beneficial impacts of agricultural inputs on maintaining soil quality, boosting productivity, and enhancing crop storage. Integrating both organic and inorganic nutrient sources can effectively improve soil fertility, leading to optimal crop yield [14].

The results suggest that joint decision-making between women and men in SWC is negatively associated with land consolidation, highlighting disparities in decision-making power within households concerning agricultural intensification. The reluctance towards joint male-female decision-making or integrating youth decisions in SWC underscores the necessity to transition towards a participatory households decision-making approach that not only accommodates women but also priorities involvement of youth. According to Ref. [60], females and youth face challenges with achieving inclusive and sustainable agricultural intensification include limited access to productive and financial resources, limited participation of women and youth in making decisions regarding SWC investments, as well as prevailing negative community attitudes, particularly towards youths. Decisions regarding sustainable agricultural intensification practices are intricately linked with other household decisions. Empowering smallholders to engage in inclusive decision making within households is important for effectively identifying synergies and trade-offs in SWC to enhance farm productivity through sustainable agricultural intensification [61]. Farmers’ reluctance towards joint household decision-making suggest the need for promoting awareness about the benefits of collective bargaining power. These findings suggest that households should operate as collective action institutions making unified decisions about SWC investments [53].

Findings that both on-farm and off-farming activities are significant for females and positively related to land consolidation. Female farmers’ preference for both livelihood activities suggests they can be classified into two groups: those who dedicate themselves fully to agriculture into a primary, allocating their labour entirely to farming, and those who diversify their income through off-farm activities, using part of those earnings to invest in sustainable agricultural intensification. Women who prioritize on-farm activities demonstrate a strong interest in participating decisions farm production and markets. According to Refs. [62,63], a significant portion of household income derived from off-farm employment leads to increased investments in farming and enhanced agricultural productivity.

The results also indicate that both the existing tenure arrangement and the new land tenure system are significant and positively linked with land consolidation. Men and women's preferences regarding land tenure rights may reflect awareness of evolving land laws aimed at ensuring equal rights to land ownership for both young women and men. This shared preference suggests a recognition by both genders of the crucial role of secured land tenure in development, advocating for secure tenure rights and equitable access to land and other resources. According to Ref. [64], inadequate land-use rights and limited women's decision-making authority over land hinder land consolidation efforts. Secure land rights and a comprehensive understanding of land issues in Rwanda serve as incentive mechanisms to enhance farm productivity and promote specialization in farming [65].

## 4 Conclusion and policy implications

The study assessed gender preferences for multiple SWC attributes, utilizing a BWS experiment survey administered to beneficiaries of the FATE project in northern Rwanda. Employing a comprehensive analysis framework combining count analysis, attribute relative importance, Pearson correlation, and the MNL model with a maximum difference, the study discovered preference heterogeneity in the relative importance of multiple SWC attribute levels. It also identified significant complementarities and trade-offs among these attribute levels, indicating that no single set of SWC attribute levels universally drives sustainable agricultural intensification. The findings underscore to expand SWC implementation and offer incentives to promote adoption of SWC practices with lower uptake rates, thereby enhancing their effectiveness. Attribute levels associated with land consolidation and crop intensification programs underscore the importance of sustainable agricultural intensification in meeting food demand amidst increasing land scarcity. Findings with MNL indicate that both males and females have positive preferences for multiple SWC practices, whereas they have negative preferences for household decision-making attribute levels. This underscores the necessity of providing equal opportunities for both males and females to achieve sustainable agricultural intensification, forming the basis for gender-equitable transformations in agriculture. As highlighted in Ref. [66], pathways to sustainable agricultural intensification vary and depend on factors such as gender dynamics, cultural norms and contexts, and institutional frameworks.

The examination of gender differences in multiple SWC attribute scenario sheds light on the transitions from the current farming system towards farm-level structural transformations, encompassing landscape changes, land consolidation, and intensified land use. The connection between sustainable agriculture intensification and farm-level structural changes has significant practical and social implications. Practically, these changes aim to reduce land degradation, increase farm yields, boost household income, and enhance household livelihoods and food security. Socially, improved food security and a sustainable environment are expected to elevate living standards among smallholder farmers and alleviate poverty.

The findings resonate with existing literature emphasizing the empowerment of smallholders in decision-making to effectively identify synergies and trade-offs in SWC aimed at sustainable intensification of agriculture. This implies the need for more strategic, medium-to long-term SWC investment decisions, focusing on key aspects of farm structural changes, mainly farm size and the degree of specialization or production intensity within the farming system.

The current study utilized cross-sectional data and the MNL model to evaluate preferences for multiple SWC attributes. The findings highlight that farmers have diverse preferences for unobserved attribute levels, while sharing common preferences for observed SWC attribute levels. However, there is notable asymmetry and heterogeneity in preferences between males and females. Further research could benefit from using BWS data and explore advanced methods such as latent class or random parameter and mixed logit. These approaches can better account for the variability in choice parameters and provide deeper insights into farmers’ decision-making processes regarding SWC measures.

## Data availability statement

Data related to this paper can be made available upon request.

## CRediT authorship contribution statement

**Ildephonse Musafili:** Writing – review & editing, Writing – original draft, Methodology, Formal analysis, Conceptualization. **Oscar Ingasia Ayuya:** Supervision. **Eliud Abucheli Birachi:** Supervision.

## Declaration of competing interest

The authors declare that they have no known competing financial interests or personal relationships that could have appeared to influence the work reported in this paper.
